# Design of Experimental Approach for Development of Rapid High Performance Liquid Chromatographic Process for Simultaneous Estimation of Metoprolol, Telmisartan, and Amlodipine from Formulation: Greenness and Whiteness Evaluation

**DOI:** 10.3390/molecules29051087

**Published:** 2024-02-29

**Authors:** Mahesh Attimarad, Mohammed Jassim Alali, Hussain Ali Alali, Dana Hisham Alabdulmuhsin, Aljohara Khalid Alnajdi, Katharigatta Narayanaswamy Venugopala, Anroop B. Nair

**Affiliations:** 1 Department of Pharmaceutical Sciences, College of Clinical Pharmacy, King Faisal University, Al-Ahsa 31982, Saudi Arabia; mhm-123-@hotmail.com (M.J.A.); hhhhussain.880@gmail.com (H.A.A.); danahesham19@gmail.com (D.H.A.); j1najdi@gmail.com (A.K.A.); kvenugopala@kfu.edu.sa (K.N.V.); anair@kfu.edu.sa (A.B.N.); 2 Department of Biotechnology and Food Science, Faculty of Applied Sciences, Durban University of Technology, Durban 4001, South Africa

**Keywords:** Box–Behnken design, chromatography, optimization, validation, anti-hypertensive, amlodipine, telmisartan, metoprolol, greenness

## Abstract

The design of an experimental approach, the Box–Behnken design, was implemented to optimize the chromatographic condition to develop a rapid HPLC procedure for quantification of a ternary mixture of metoprolol (MET), telmisartan (TEL), and amlodipine (AML) from the formulation. The perturbation plots, contour, and 3D response surface pictures were developed to study the impact of each variable on the analytes’ retention time and the probable interaction between the parameters with fewer chromatographic runs. The optimized HPLC method separated the three analytes within 5 min with excellent selectivity and peak shape on a Zorbax C18 HPLC column using acetonitrile and phosphate buffer (20 mM, pH 5.8) with isocratic elution at a 1.1 mL/min flowrate. A wavelength 230 nm was utilized to monitor the elute. The validation of proposed method demonstrated a wide linearity range of 10–200 µg/mL for MET and TEL and 5–50 µg/mL for AML along with an excellent correlation coefficient. The correctness of the HPLC approach was further confirmed by excellent recovery of the added amount of analytes utilizing the standard addition technique. The recommended HPLC approach was employed safely for quality assurance of the formulation, because the evaluation of the method’s greenness and whiteness confirmed the environmentally friendly nature of the approach.

## 1. Introduction

**Figure 1:**
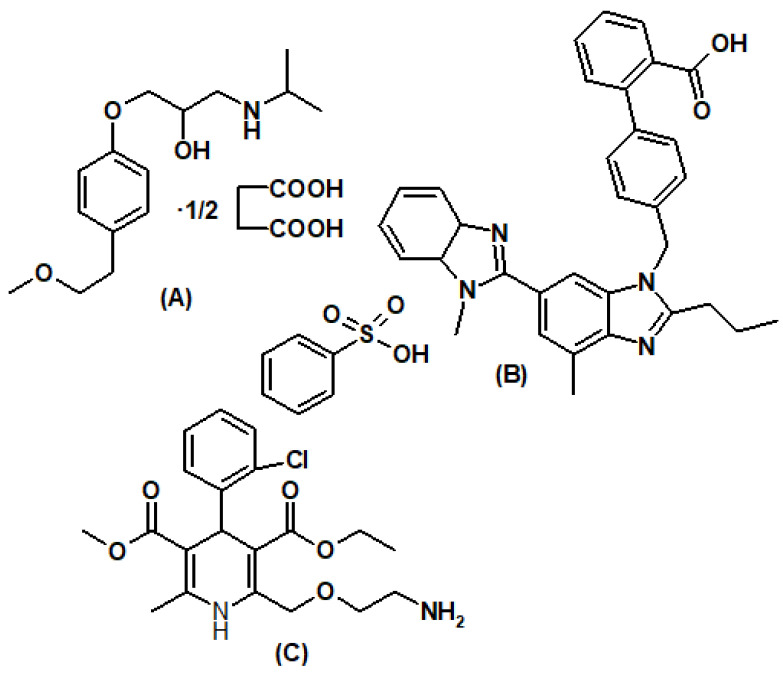
Chemical structure of metoprolol succinate (**A**), telmisartan (**B**), and amlodipine besylate (**C**).

High blood pressure is one of the prominent factors for various cardiovascular complications leading to death [1]. Monitoring normal blood pressure is very important to reduce cardiovascular complications and chest pain due to angina pectoris. Hence, a new combination of metoprolol, telmisartan, and amlodipine with different mechanisms of action has been developed for the management of hypertension and angina. The different analytes of fixed-dose combos have distinct mechanisms of action; they quickly lower blood pressure compared to monotherapy and lower the risk of cumulative events related to cardiovascular disease. These benefits translate into 2–5 times higher antihypertensive effects. Combination therapy also improves treatment compliance, reduces the risk of treatment failure, minimizes adverse effects, and enhances the protection of delicate organs [2]. Metoprolol succinate (MET, Figure 1A) in low doses (less than 100 mg) selectively inhibits beta-1 adrenergic receptors, thereby reducing the heart rate, cardiac output, and oxygen requirement of the heart muscle [3]. MET lowers cardiac output by competitively blocking catecholamine function at peripheral adrenergic neurons. It also lowers peripheral sympathetic circulation and lowers renin expression. MET lowers the oxygen requirements of the heart at any given degree of effort by preventing catecholamine-induced increases in heart rate, blood pressure, and the rate and intensity of myocardial contraction. This property makes MET beneficial in the persistent care of angina pectoris. Telmisartan (TEL, Figure 1B) inhibits the angiotensin 2 type 1 (AT1) receptor precisely and inconvertibly, and the pressor action of angiotensin 2 on the heart, blood vessels, and kidney is blocked. Because the harmful consequences of angiotensin II, such as blood vessel vasoconstriction, organ inflammation, and cardiac mitochondrial malfunction, are triggered by the activation of angiotensin II type 1, the smooth muscles of the heart and blood vessels are relaxed, which reduces the blood pressure [4]. Since TEL can inhibit angiotensin II type 1, it also has cardio-protective effects by lowering the production of reactive free radicals. Amlodipine besylate (AML, Figure 1C), a dihydropyridine derivative, was the first calcium channel blocker to be used to lower elevated blood pressure and relieve chest discomfort. AML produces its effect by increasing the transportation of calcium ions into the cardiomyocytes. This increases the diameter of the blood vessels by relaxing the smooth muscles of blood vessels, which reduces blood pressure and facilitates the additional blood supply to cardiac muscle cells. This decreases the work pressure of the heart and reduces pain in the chest [5]. Analytical procedures are well documented in the literature for the analysis of MET, TEL, and AML alone and along with other drugs [6,7,8,9,10,11,12,13]. The literature documented the implementation of the HPLC technique [14] for the quantification of AML along with its impurities in formulation. RP HPLC [15], UPLC [16], LC-MS [17,18,19], and capillary electrophoresis [20] methods are reported for the analysis of AML along with TEL from the formulations. Yenduri et al. [21] compared the greenness of several HPLC methods documented for the concurrent assessment of TEL and AML. The literature documented the implementation of UV spectroscopy [22] and HPLC [23] techniques for the quantification of TEL in formulation and plasma, respectively. UV spectroscopic [24], UV derivative [25], HPTLC [26], and spectrofluorometric [27] approaches are described for the quantification of AML and MET. The analysis of MET was performed using UV spectroscopic and HPLC methods from formulation and plasma [28,29]. HPTLC [30] and LC-MS [31] approaches are explained for analysis of TEL and MET. As the HPLC method for quality control for this tri-mixture formulation has not been documented in the literature, we developed a rapid, accurate, and straightforward reverse phase HPLC method in this work using multivariate optimization to simultaneously quantify AML, TEL, and MET from the formulation.

Quality by design (QbD) is an essential step in the product development process, as per the FDA requirements for understanding the effect of the variable parameters on the manufacturing process [32]. Hence, for the development of reliable and robust analytical methods for quality control of formulations, the QbD approach has been extended, known as analytical QbD (AQbD) [33]. Design of experiment (DoE) is the crucial element of AQbD [34]. DoE utilizes a scientifically driven principle to identify the combined effect of all independent variable constraints on the dependent variables in the development of HPLC methods [35]. The conventional univariate method is based on a trial-and-error approach because it involves changing one chromatographic parameter at a time until all chromatographic parameters are optimized and all chromatographic peaks are resolved with good peak shape and asymmetry. Further, the development of a robust analytical method is difficult using univariate methods and utilizes more time and resources. Hence, to develop a robust HPLC method, it is important to study the interaction of all independent variable parameters responsible for final analysis. The full factorial design utilizes a multivariate approach and provides the combined effect of all variable parameters [36,37,38,39,40,41]. In addition, multivariate analysis allows conclusions to be formed across numerous outcomes and can produce estimates for individual outcomes that are more accurate and less biased. Additionally, it permits the simultaneous assessment of several variables and results, offering a broader awareness of the research subject matter. An experimental design type called a Box–Behnken design (BBD) is adopted to optimize chromatographic techniques. It has several advantages, such as optimization of the chromatographic conditions with fewer analysis runs with many independent variables, saving time and resources [42,43,44]. Hence, we utilized the BBD approach to develop a robust HPLC method to predict the optimized chromatographic conditions using the design space/design region and exploit for quality control of formulation.

## 2. Materials and Methods

### 2.1. Materials

The reference standards amlodipine, telmisartan, and metoprolol were purchased from Biochemix India Limited (Hyderabad, India). Acetonitrile of HPLC grade was bought from Sigma Aldrich. Potassium dihydrogen phosphate and dipotassium hydrogen phosphate used for the preparation of buffer were analytical-grade and acquired from Merck. The ortho-phosphoric acid used for adjusting the buffer pH was procured from Fine Chemicals. The amlodipine besylate (5 mg) capsule, telmisartan (40 mg), and metoprolol succinate (50 mg) tablets were purchased at the neighborhood store.

### 2.2. Instrument

High-performance liquid chromatography (Agilent Technologies, 1200 series, Waldbronn, Germany) attached with an auto sampler, a binary pump with the four solvents option, a degasser, and a UV-Vis detector was utilized for performing the experiments. Three active ingredients were separated on the Zorbax C18 RP HPLC column (150 mm length, 4.6 mm i.d, 5 µm particle size (Agilent technologies, Theale, UK). Samples were weighed using a digital balance with four decimals (Shimadzu, Kyoto, Japan) and the mobile phase’s pH was attuned by means of a pocket-sized digital pH meter (Martini instruments, Gallarate, Italy).

### 2.3. Preparation of Mobile Phase

The phosphate buffer (20 mM, 5.8 pH) was prepared by mixing 18.3 mL of 1 M potassium dihydrogen phosphate (13.61 g/100 mL) and 1.7 mL of 1 M potassium dihydrogen phosphate (17.41/100 mL) solutions into a final volume of 1000 mL water. The buffer solution and HPLC-grade acetonitrile were sonicated for 30 min and filtered through 0.45-micron Whitman filter paper before use for degassing.

### 2.4. Preparation of Reference Standard Solutions

Amounts of 100 mg of metoprolol, telmisartan, and amlodipine were weighed individually, and subsequently placed into dry, clean, 100 mL volumetric flasks. Acetonitrile was utilized to dissolve the pure active ingredients. To completely dissolve, the telmisartan solution was sonicated for fifteen minutes. The HPLC mobile phase was used to further dilute the stock solutions, maintaining the 45% of acetonitrile and 55% of buffer solution in order to generate the calibration curve.

### 2.5. Box–Behnken Model to Optimize Chromatographic Conditions

Based on the preliminary studies previously carried out on these active ingredients in our laboratory and based on literature reports of optimization, three factors (pH of the buffer, flow rate, and percentage of acetonitrile) were employed to optimize the chromatographic constraints [6,8]. With the help of the Box–Behnken model, the three independent constraints at three points were optimized. The 17 conditions (Table 1) that the Design Expert program (Version 12.0.3.0) recommended were carried out randomly. The three conditions for the three factors were 3.0, 4.5, and 6.0 pH of the buffer (A); 35%, 40%, and 45% of acetonitrile (B); and 0.8, 1.0, and 1.2 mL/min flow rate (C). All three analytes’ retention times (Y) were chosen as the response. The interaction of three factors is expressed in terms of quadratic polynomial equations (Equation (1)) to predict the optimized chromatographic conditions using the design space.

Y = δ_0_ + δ_1_A + δ_2_B + δ_3_C + δ_4_A^2^ + δ_5_B^2^ + δ_6_C^2^ + δ AB + δ_8_ AC + δ_9_ BC (1)

where δ_0_ is the intercept; δ_1,_ δ_2,_ and δ_3_ are coefficients of three variable parameters; δ_4,_ δ_5,_ and δ_6_ are coefficients of quadratic functions of three variable parameters; δ_7,_ δ_8,_ and δ_9_ are coefficients of the combined effect of two variable parameters.

Further, various ANOVA parameters such as *p*-values (*p* < 0.05), coefficient of determination (R^2^), and Adeq Precision (more than 4) were calculated. Additionally, the response surface methodology was applied to create 3D response surface designs, contour illustrations, and perturbation sketches to examine the impact of each variable on the analytes’ retention time and to determine the probable interaction between the parameters. The optimized chromatographic condition was predicted using the desirability function. A design space plot showing optimal chromatographic conditions was also constructed using numerical and graphical representation.

### 2.6. Calibration Curve Construction

A micropipette was utilized to transfer an appropriate amount of reference standard solutions of AML, TEL, and MET into a 1.5 mL vial. The total volume was then made to 1 mL using acetonitrile and phosphate buffer, maintaining the mobile phase composition to obtain amounts of 5, 10, 20, 30, 40, and 50 µg/mL of AML, and 10, 40, 80, 120, 160, and 200 µg/mL of TEL and MET. Amounts of 20 µL of each of the three analyte working standard solutions were injected into the HPLC instrument. The Zorbax C18 RP HPLC column was utilized for the separation of the analytes under optimal chromatographic conditions, which included isocratic elution with acetonitrile 45% + 20 mM phosphate buffer 55% (pH 5.8) at room temperature. Eluent was examined at 230 nm by means of a diode array detector, while the mobile phase was propelled at 1.1 mL per minute and the chromatogram was recorded using ChemStation software (Agilent Technologies, Waldbronn, Germany, Ver. B.04.03-SP1). The peak area of the chromatogram was determined after auto integration and plotted against the corresponding concentration of all three analytes to construct a linearity relationship, regression equation, and correlation coefficient.

### 2.7. Sample Preparation for the Analysis of Formulation

There was no formulation readily accessible in the neighborhood that included all three of the active components. Hence, the 20 tablets of telmisartan (40 mg) and metoprolol succinate (50 mg) per tablet powder were mixed with the 20 tablets of amlodipine (5 mg) powder. The blend of all three active components corresponding to 4 mg of telmisartan, 5 mg of metoprolol succinate, and 500 µg of amlodipine was shifted to a 10 mL graduated flask and acetonitrile was added to obtain a clear solution through sonication. The mixture was filtered by employing Whatman filter paper, and then enough mobile phase was added to the clear filtrate to obtain the desired quantity of analytes within the calibration concentration range.

### 2.8. Validation of the HPLC Method

To ensure that the designed analytical approach is appropriate for the formulation’s quality control, the analytical method must be validated. Therefore, using the ICH Q2 (R1) guidelines, the suggested HPLC technique was verified for linearity, limit of detection and quantification, system adaptability, correctness, precision, and robustness study.

#### 2.8.1. System Suitability Study

An analysis of a mixture of AML (10 µg/mL), TEL (80 µg/mL), and MET (100 µg/mL) under optimum chromatographic conditions was carried out as part of the system suitability research. Following six replications, the percentage relative standard deviation (%RSD) for peak asymmetry, retention time, peak area, and theoretical plate was determined.

#### 2.8.2. Linearity and Sensitivity

A linear relationship was established for all three analytes by determining the peak area of the chromatogram contrary to the corresponding concentration of MET (10–200 µg/mL), TEL (10–200 µg/mL), and AML (5–50 µg/mL). The linear regression equation was constructed using the above data and the correlation coefficient was also determined. The limit of detection (LOD) and limit of quantification (LOQ) calculations were used to assess the method’s sensitivity. LOD and LOQ were determined by means of 3.3 and 10 times the standard deviation of the intercept to the slope, respectively.

#### 2.8.3. Correctness and Precision

The recommended RP HPLC method’s correctness was determined by evaluating all three analytes in three different concentrations (low QC, medium QC, and high QC) within the complete calibration range. The mixture of 10, 100, and 200 µg/mL each of MET and TEL and 5, 25, and 50 µg/mL of AML was evaluated three times, and % RSD and percentage relative error (%RE) were calculated. Less than 2% of %RSD and less than ±2% %RE are acceptable as per the ICH guidelines. The precision of the approach was evaluated in terms of both within- and between-day precision. The above solutions were examined three times on one day for within-day precision and three times on successive days for between-day precision. The precision of the procedure is presented in percentage assay.

#### 2.8.4. Robustness

A robustness study was performed to determine the impact of insignificant but considerable variations in chromatographic conditions on the peak area. The investigation was executed by altering the pH of the mobile phase (±0.1), acetonitrile concentration (±2 mL), flow rate (±0.1 mL), and wavelength (±1 nm). The peak areas of three analytes were estimated by analyzing the mixture of MET (100 g/mL), TEL (80 g/mL), and AML (10 g/mL), via altering one parameter at a time while maintaining the other chromatographic conditions ideal.

### 2.9. Application of HPLC Method to Formulation

For the analysis, 20 µL of sample solution was administered into the HPLC instrument and examined under the optimal chromatographic situation, with the concentration determined using relevant regression equations. The accuracy of the proposed HPLC method was further validated using the standard addition technique. The previously examined formulation solution was re-analyzed after adding a known amount of each of the three analytes at three levels, the total amount of analytes was determined using the relevant regression equations, and the percentage recovery of the amount that was added was computed. The accuracy is given as a mean percentage recovery and a percentage relative error.

## 3. Results and Discussion

All three analytes were polar molecules; hence, the reverse phase HPLC method was developed using the Zorbax C_18_ column. To develop a rapid HPLC method, a 150 mm column was selected. For the selection of analysis wavelength, overlapped UV spectra of MET, TEL, and AML were studied; TEL showed very good absorption in the range of 200–300 nm, whereas MET and AML showed an isosbestic point at 230 nm; hence, the wavelength of 230 nm was chosen for further chromatographic optimization.

### 3.1. The Optimization of Chromatographic Parameters

To develop the robust HPLC method for concurrent estimation of MET, TEL, and AML, the chromatographic conditions were optimized using the AQbD approach. The different independent variables that could be effective constraints for the establishment of a robust HPLC technique were the pH of the buffer solution, molar strength of the buffering agent, percentage of organic modifier, flow rate of the mobile phase, HPLC column temperature, and injection volume of the sample. As the formulation contains a high amount of analytes, 20 µL of sample injection was sufficient for the quantification of all three analytes. In order to analyze the formulation at room temperature, an analytical method was developed at ambient temperature. The previous HPLC methods developed in our laboratory for the quantification of the same class of antihypertensive drugs revealed that the molar strength of buffer salt showed negligible effect on the separation of analytes. In contrast, the mobile phase pH, flow rate, and organic phase ratio all had a significant influence [6,8]. Hence, for the optimization of these three parameters, the Box–Behnken design (BBD) was adopted. The BBD is a rotatable full factorial optimization design requiring 2γ(γ − 1) + n experiments where γ are several independent variables at three levels (−1, 0, +1), that is, 12 experiments plus n center points. However, the software proposed 17 experiments, including five center points, which were carried out in random order to eliminate bias. Further, coded quadratic polynomial equations for the responses Y_1_, Y_2_, and Y_3_ to retention times for MET, TEL, and AML, respectively (Table 1), were used as per the software suggestion to predict the cumulative influence of independent factors on the retention time of the analytes. Initially, it was planned to use the resolution factor values as the response; however, the positions of TEL and AML peaks were exchanged during some HPLC runs, and hence the retention time was used as the response. However, retention factor valves were also calculated for all runs (Supplementary Materials Table S1)

Y_1_ = +1.76 + 0.5204 A − 0.1751 B − 0.4075 C − 0.0725 AB − 0.1138 AC + 0.0503 BC + 0.3701 A^2^ + 0.0586 B^2^ + 0.0659 C^2^

Y_2_ = +18.79 − 0.7724 A − 7.59 B − 7691 C + 0.355 AB + 0.119 AC + 2.31 BC – 13.07 A^2^ + 0.9313 B^2^ +0.0055 C^2^

Y_3_ = +3.96 +2.36 A − 1.98 B − 1.12 C − 1.52 AB − 0.5228 AC + 0.4823 BC + 1.91 A^2^ + 1.10 B^2^ − 0.1362 C^2^

where A is the pH of the buffer, B is the percentage of acetonitrile, and C is the flow rate. It is clear from the signs of the coefficients that the pH of the buffer had a detrimental impact on the retention time of the MET (Y_1_) and AMD (Y_3_) and an advantageous influence on the retention time of TEL (Y_2_). The percentage of acetonitrile and flowrate had a negative effect on all three analytes’ retention times. The quadratic functions of all three factors had a negative influence on MET retention time, whereas the combined effect of variables had a favorable effect on the retention time of TEL. The cumulative influence of pH with flowrate and acetonitrile concentration showed a negative effect, whereas flowrate and acetonitrile showed a positive effect on Y_1_ and Y_3_.

Further, the quadratic model was validated by an ANOVA statistical study. The predicted R^2^ valves for the retention time of MET and AMD were very close to the adjusted R^2^ valves; the difference was less than 0.2, whereas the predicted R^2^ value for TEL showed a negative valve, indicating that a mean value or higher-order model can predict better. However, the Adeq precision values for all three responses (54.52, 8.50, and 106.73 for Y_1_, Y_2_, and Y_3_, respectively) were more than 4, indicating that a sufficient signal can be applied to circumnavigate the design space. The high value shown for the Y_1_ (243.74), Y_2_ (5.69), and Y_3_ (797.51) model F value indicates that the model is substantially important. Further, there is a 0.01% possibility in the case of Y_1_ and Y_3_ and a 0.37% chance in the case of Y_2_ that the high F value is caused by noise. Most of the *p*-values for the responses were below 0.05, indicating that model expressions are significant.

**Figure 2:**
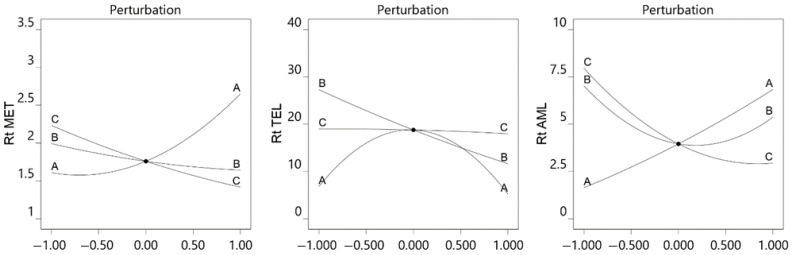
Perturbation plot for effect of pH of buffer (A), acetonitrile concentration of mobile phase (B), and flow rate (C) on retention time of MET, TEL, and AML.

The perturbation plot represents the effects of all the studied parameters on the single graph. It depicts the changes in the response with changes in the individual variables from the center point. Perturbation plots (Figure 2) showed that an increase in the pH and decrease in the flow rate increased the retention time of MET and AMD, whereas TEL showed very high retention at pH 4.5, and the retention time was less for TEL at pH 3 and 6. The MET and TEL eluted more quickly when the mobile phase’s acetonitrile fraction increased. Flow rate had a negligible influence on TEL retention time, whereas the buffer pH and acetonitrile concentration had a significant impact.

**Figure 3:**
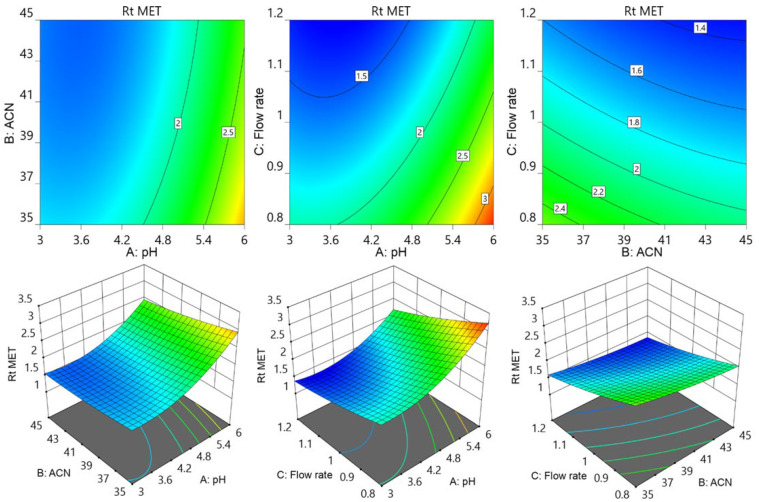
Contour and surface response models for showing effect of pH of buffer (A), acetonitrile percentage of mobile phase (B), and flow rate (C) on retention time of MET.

**Figure 4:**
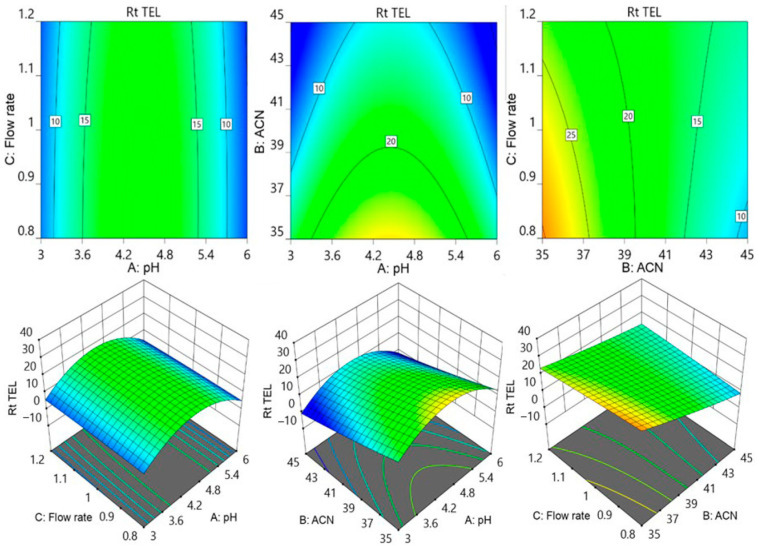
Contour and surface response models for showing effect of pH of buffer (A), acetonitrile concentration (B), and flow rate (C) on retention time of TEL.

**Figure 5:**
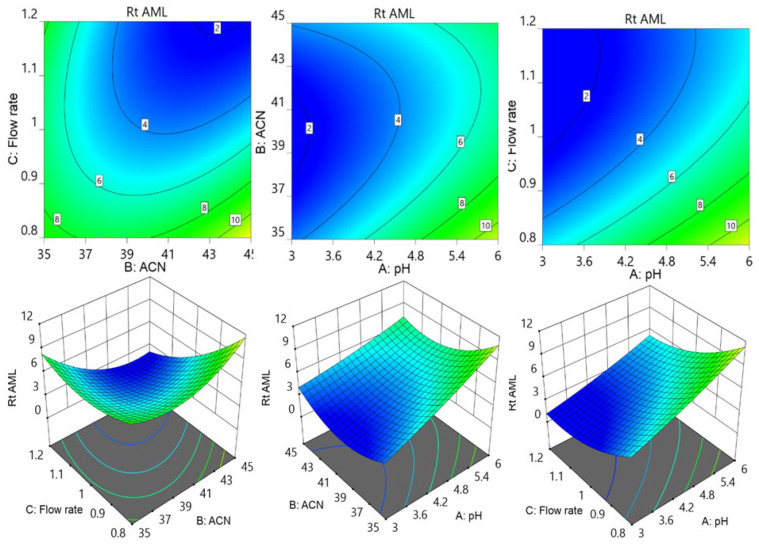
Contour and surface response models for showing effect of pH of buffer (A), acetonitrile concentration (B), and flow rate (C) on retention time of AML.

Further contour and surface response diagrams (Figure 3) were studied to display the combined influence of independent factors on the response. The contour plot illustrates the two-dimensional relationship between one dependent variable and two independent variables. The X and Y axes are used to indicate the independent variables (pH of the mobile phase, flow rate, or acetonitrile concentration), while contour lines and bands are used to represent the dependent variable (analyte retention time). The contour lines establish connections between the combined impacts of independent variables on the response. The contour design exhibited a curvilinear relationship, indicating that the parameters have a nonlinear outcome on the analytes’ retention time. The retention period of MET was extended by an increase in pH accompanied by a reduction in acetonitrile concentration as well as a decrease in flow rate. The retention period of MET was lowered by increasing the flow speed in conjunction with an increase in acetonitrile. The collective influence of flow rate with pH and with acetonitrile concentration showed an inverted parabolic impact on TEL’s retention point. The TEL retention period was nonlinearly decreased by the drop in flow rate and acetonitrile concentration (Figure 4). It became apparent that the combined effects of the acetonitrile concentration with flowrate and with buffer solution pH had opposing effects on the AML retention time. The retention time of AML jumped when the percentage of acetonitrile dropped with a reduction in flow rate; however, the retention time of AML reduced when the pH of the buffer decreased. The retention duration of the AML was prolonged by a decrease in flow rate as the pH of the buffer was raised (Figure 5).

**Figure 6:**
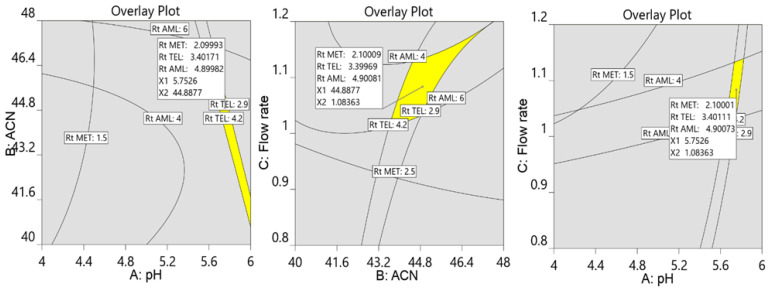
Overlay plot of design space displaying the optimized chromatographic conditions for separation of MET (2.1 min), TEL (3.4 min), and AML (4.9 min). The value of 5.75 is pH of the buffer, 44.88% is percentage of acetonitrile, and 1.08 mL/min is flow rate.

Further, the numerical and graphical optimization criteria were used to select the optimum chromatographic conditions via trading off designated answers. The objective of the numerical optimization in this work was to minimize the retention time along with good resolution between the analytes, with a desirability close to 1 to develop the robust chromatographic method. In order to illustrate the design space where all three factors appear, graphic optimization was also employed. Equal resolution for all three analytes was the aim of the graphical optimization; hence, the retention times for MET, TEL, and AML were set at two minutes, 3.5 min, and five minutes, respectively. Finally, the design space with a yellow area was displayed, consisting of two variables keeping one variable at a constant value (Figure 6).

**Figure 7:**
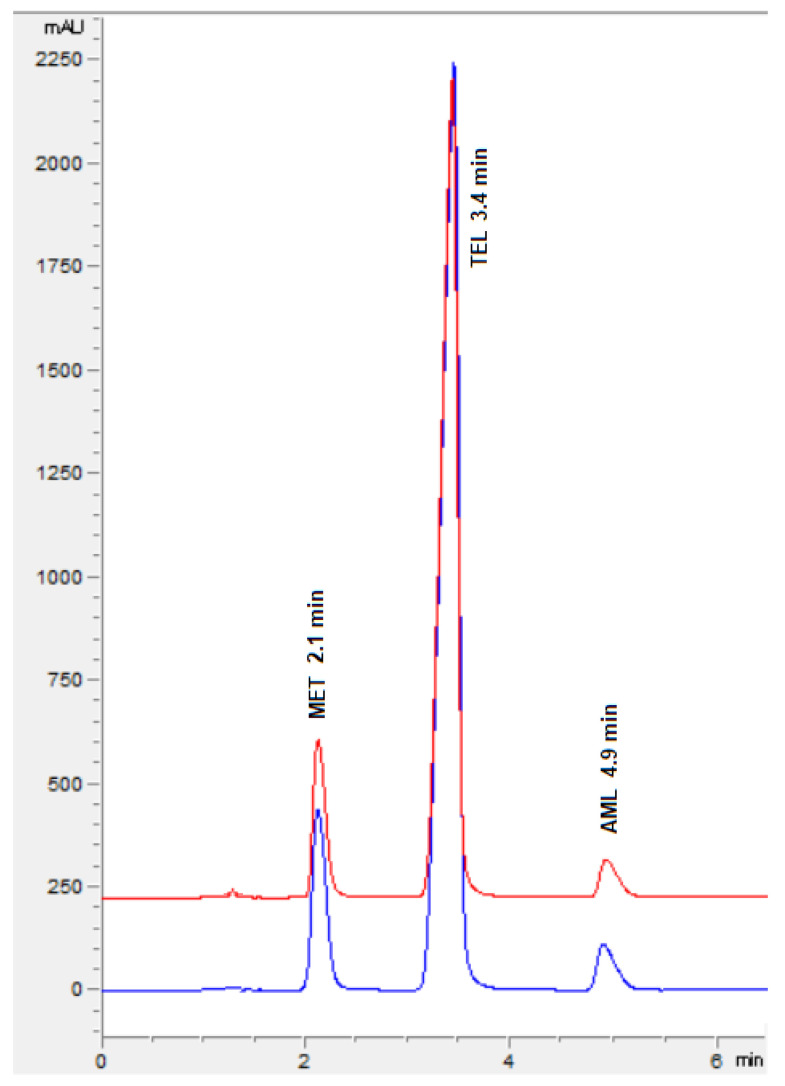
Representative chromatogram of MET, TEL, and AML for standard solution (blue) and formulation (red).

The ideal chromatographic conditions, as indicated by Design Expert, include 45% acetonitrile, 55% phosphate buffer (20 mM) with pH 5.8, and 1.1 mL/min of flow speed with a desirability of 1. The experimental model was authenticated by performing the experiments and adopting the optimized chromatographic conditions, and the observed retention times agree with the predicted retention times along with a percentage relative error of less than ±5% (Figure 7). This confirmed the ability of the design space to predict the optimal experimental conditions.

### 3.2. HPLC Method Validation

#### 3.2.1. System Suitability Analysis

A system suitability investigation was implemented to confirm that the theoretical plate, resolution, capacity factor, tailing factor, and repeatability of the chromatographic analysis findings are adequate for the quality control for the formulation. The resolution, tailing factor, capacity factor, and theoretical plate were in the acceptable range along with a low percentage relative standard deviation (Table 2).

#### 3.2.2. Linearity and Sensitivity

All three analytes were analyzed using optimized chromatographic conditions; all three analytes demonstrated a substantial correlation among concentration and peak area within the formulation’s linearity concentration range. AMD showed good linearity within the 5–50 µg/mL region, while MET and TEL showed linearity within the 10–200 µg/mL range and the correlation coefficient was at an acceptable level (R2 > 0.999) (Supplementary Materials Figures S1 and S2). The slope, intercept, and correlation coefficients were tabulated (Table 2). Utilizing the optimized chromatographic conditions, all analytes in the formulation could be quantified due to the high detection and quantification limits (Table 2).

#### 3.2.3. Correctness and Precision

The between-day and within-day correctness and precision were determined by performing the three replications of HPLC analysis using optimized chromatographic conditions on three different concentrations of analytes. The calculated percentage RSD was in the range of 0.99–1.91 for between-day and 1.19–1.77 for within-day precision. The RSD of less than 2% for both between-day and within-day precision confirmed that the suggested HPLC approach is repeatable and appropriate for a quality control study of the formulation. The assay findings ranged from 98.18 to 100.80% and 98.38 to 101.30% for between-day and within-day precision, respectively, along with a percentage relative error of less than ±2, confirming the correctness of the HPLC approach (Table 3).

#### 3.2.4. Robustness

By evaluating the mixture of MET, TEL, and AML and adjusting one constraint at a time, although holding the other chromatographic conditions at optimal levels, the robustness of the suggested HPLC approach was ascertained. The HPLC method’s robustness was confirmed when the robustness, given as a percentage RSD of the peak area, was determined to be around 2% (Table 4).

### 3.3. Analysis of Formulation

In the presence of formulation excipients, the optimized HPLC technique was utilized for the assessment of MET, TEL, and AML without separation. The amount of MET, TEL, and AML was 50.37 mg, 39.56 mg, and 4.96 mg per tablet mixture, which were in agreement with the formulation amount. Additionally, the accuracy was verified using the standard additive technique, yielding average percentage recoveries of 98.46%, 98.51%, and 99.43% for the added amounts of MET, TEL, and AML, respectively, along with less than 2% of RSD and less than ±2% of relative error. It attested the accuracy of the approach and the noninterference from the formulation’s excipients (Table 5). For the quality control of formulations including MET, TEL, and AML, the suggested HPLC approach can therefore be applied.

### 3.4. Assessment of Greenness and Whiteness of the HPLC Method

**Figure 8:**
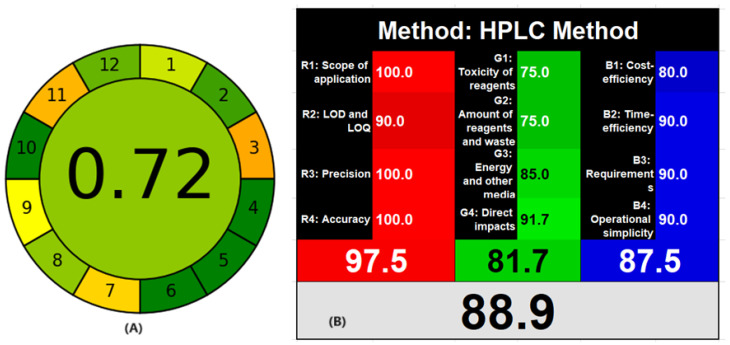
AGREE (**A**) and white analytical chemistry whiteness (**B**) evaluation report for the proposed HPLC method.

Green analytical chemistry is an impression to be considered during the development of analytical methods for the health and safety of animals and the environment. Different quantitative evaluation techniques are available for the determination of the greenness of the analytical methods. Using two different greenness evaluation techniques, the Analytical GREEness matric approach (AGREE) and white analytical chemistry, the proposed HPLC method was assessed. AGREE is a freely available software for gauging the greenness of the HPLC approach utilizing the 12 green analytical principles [45]. All 12 principles are scored from 0 to 1 for dangerous to safe (red–orange–yellow–green), along with a flexible weightage for each criterion. The greenness of the analytical method was represented as a pictorial circle with all 12 principles scored at the periphery of the circle clockwise and the overall score at the center. The proposed HPLC method showed an overall score of 0.72, confirming the safe-to-use assay technique for the repetitive systematic quality control of the formulation (Supplementary Materials Figure S3). As the three analytes were separated within 5 min, using a small amount of mobile phase generates less waste. Further, the mobile phase consists of only 45% of organic solvent. White analytical chemistry [46] also utilizes 12 green analytical principles along with the practical applicability and quality of the analytical method known as 12 white analytical principles. The 12 analytical principles are divided into three clusters (green–red–blue) with four constraints for each cluster. The green color describes the volume, toxicity of reagents and amount of waste produced, total energy used, and how much they affect the health of the animal. The red color illustrates the scope of the analytical method and certification factors such as LOD, LOQ, accurateness, and reproducibility. The blue color represents the economic aspect, time required, training, instrument, and manpower requirements. The overall outcome is presented as a combination of all three colors into whiteness. The proposed HPLC method showed 88.9 (Supplementary Materials Figure S4) as the method has a very high scope, because the quantification of three analytes using UV spectroscopy is difficult. Further, the method was highly accurate and precise with high sensitivity. As the method was rapid, less waste was generated per sample analysis along with time and cost efficiency. The use of green solvents such as ethanol is an alternative to acetonitrile; however, the back pressure is high with ethanol, requiring a stronger quaternary pump to withstand the back pressure or UPLC system. As a result, drug testing organizations as well as the medical industry can employ the suggested HPLC technique for consistent quality assurance of MET, TEL, and AML preparations using traditional HPLC systems. Further, the score of WAC is higher than the AGREE due to the inclusion of scope, quality of analytical method, and practical requirements (Figure 8).

## 4. Conclusions

The chromatographic conditions for the establishment of the RP HPLC technique for the concurrent quantification of MEL, TEL, and AML from the ternary composition were predicted using the DoE-based Box–Behnken design. The perturbation plots, contour diagrams, and 3D response surface pictures demonstrated the impact of each variable on the analytes’ retention time and the probable interaction between the parameters with fewer chromatographic runs. Further, the selected numerical and graphical optimization criteria suggested accurate chromatographic conditions using the design space with a desirability of 1. The baseline separation of all three analytes’ peaks with sufficient resolution and excellent peak shape was accomplished using the predicted HPLC conditions within 5 min. The evaluation of greenness and whiteness of the proposed HPLC method by AGREE and white analytical chemistry, respectively, confirmed the ecofriendly nature of the method. Additionally, the verified analytical approach was straightforward, perfect, exact, rapid, and robust; as a result, it was successfully applied for quality control of formulations containing MET, TEL, and AML.

## Figures and Tables

**Table 1:** Box–Behnken design for MET, TEL, and AML HPLC method with response.

		X1	X2	X3	Y1	Y2	Y3
Standard	Run	A:pH of Buffer	B:Acetonitile Percent	C:Flow Rate	Rt MET	Rt TEL	Rt AML
12	1	4.5	45	1.2	1.386	9.485	2.38
5	2	3	40	0.8	1.982	7.756	3.909
14	3	4.5	40	1	1.756	19.012	3.89
1	4	3	35	1	1.792	10.794	5.204
10	5	4.5	45	0.8	2.03	5.68	3.569
6	6	6	40	0.8	3.295	5.914	9.872
16	7	4.5	40	1	1.764	18.697	3.96
17	8	4.5	40	1	1.771	18.567	4.012
4	9	6	45	1	2.44	3.199	5.685
3	10	3	45	1	1.589	3.985	4.216
2	11	6	35	1	2.933	8.606	12.748
9	12	4.5	35	0.8	2.483	34.567	8.42
11	13	4.5	35	1.2	1.638	29.112	5.302
15	14	4.5	40	1	1.759	18.96	3.97
13	15	4.5	40	1	1.749	18.69	3.959
8	16	6	40	1.2	2.182	3.902	6.508
7	17	3	40	1.2	1.324	5.265	2.636

MET: metoprolol; TEL: telmisartan; AML: amlodipine.

**Table 2:** Validation parameter results for MET, TEL, and AML by HPLC method.

HPLC Parameters	MET	TEL	AML	Acceptable Value
Retention time (min) ± SD	2.15 ± 0.03	3.48 ± 0.02	4.98 ± 0.04	-
Resolution ± SD	-	6.52 ± 0.028	5.96 ± 0.019	>2
Capacity factor	1.1	2.45	3.97	>1
Theoretical plate ± SD	2452.45 ± 21.14	3551.70 ± 42.57	4672.01 ± 58.64	>2000
Linearity range (µg/mL)	10–200	10–200	5–50	-
Slope	21.382	135.75	27.678	-
Intercept	−12.91	−179.67	−11.18	-
Correlation coefficient (R^2^)	0.9999	0.9999	0.9998	-
LOD (µg/mL)	1.21	2.60	0.11	-
LOQ (µg/mL)	3.68	7.88	0.33	-

MET: metoprolol; TEL: telmisartan; AML: amlodipine; SD: standard deviation.

**Table 3:** Correctness and precision results of proposed HPLC method.

		Within-Day					Between-Day		
	Amount of Drug [µg/mL]	Amount Found Mean [n = 3] ± SD	% RSD	% Recovery	% RE	Amount Found Mean [n = 9] ± SD	% RSD	% Recovery	% RE
MET	10.00	9.95 ± 0.19	1.91	99.50	−0.50	9.89 ± 0.17	1.73	98.90	−1.10
	100.00	100.48 ± 1.45	1.44	100.48	0.48	98.45 ± 1.75	1.77	98.45	−1.55
	200.00	197.85 ± 2.14	1.07	98.93	−1.08	196.95 ± 3.14	1.58	98.48	−1.53
TEL	10.00	9.94 ± 0.16	1.61	99.40	−0.60	10.13 ± 0.12	1.19	101.30	1.30
	100.00	98.67 ± 1.79	1.81	98.67	−1.33	99.07 ± 1.69	1.70	99.07	−0.93
	200.00	198.03 ± 2.92	1.47	99.02	−0.98	198.55 ± 3.46	1.74	99.28	−0.72
AML	5.00	5.04 ± 0.05	0.99	100.80	0.80	4.96 ± 0.08	1.63	99.20	−0.80
	25.00	24.63 ± 0.37	1.50	98.52	−1.48	24.68 ± 0.43	1.69	98.72	−1.28
	50.00	49.09 ± 0.86	1.75	98.18	−1.82	49.19 ± 0.86	1.75	98.38	−1.62

MET: metoprolol; TEL: telmisartan; AML: amlodipine; SD: standard deviation; %RSD: percentage relative standard deviation; %RE: percentage relative error.

**Table 4:** Robustness results for proposed HPLC method.

		Average Peak Area ± SD		
Parameters	Conditions	MET	TEL	AML
mobile phase pH	5.75 (−0.05)	2135.78 ± 9.54	10,564.85 ± 88.38	268.59 ± 3.47
	5.8 pH	2126.45 ± 12.15	10,681.47 ± 91.07	266.11 ± 5.62
	5.85 (+0.05)	2142.59 ± 8.37	10,576.43 ± 79.66	260.34 ± 4.05
	%RSD	0.38	0.61	1.60
Acetonitrile concentration	43% (−2%)	2118.97 ± 8.64	10,594.55 ± 65.37	257.96 ± 2.35
	45%	2126.45 ± 6.73	10,681.47 ± 95.67	266.11 ± 3.78
	47% (+2%)	2131.34 ± 8.96	10,613.64 ± 83.55	261.47 ± 6.14
	%RSD	0.29	0.43	1.56
Wavelength (nm)	229 (−2)	2169.63 ± 11.47	10,712.38 ± 45.63	258.15 ± 3.18
	230 nm	2126.45 ± 8.68	10,681.47 ± 57.04	266.11 ± 4.09
	231 (+2)	2095.86 ± 4.83	10,624.24 ± 37.58	271.75 ± 2.98
	%RSD	1.74	0.42	2.58
Flow rate mL/min	1 (−1)	2156.57 ± 12.75	10,908.36 ± 120.47	272.17 ± 3.55
	1.1 mL/min	2126.45 ± 10.68	10,681.47 ± 107.25	266.11 ± 5.92
	1.2 (+1)	2097.46 ± 7.92	10,475.62 ± 104.67	258.38 ± 4.73
	%RSD	1.39	2.03	2.60

MET: metoprolol; TEL: telmisartan; AML: amlodipine; SD: standard deviation; %RSD: percentage relative standard deviation.

**Table 5:** Assay results of formulation and standard addition method.

Formulation	Amount of Drug [mg/Tab]	Amount Found Mean [n = 3] ± SD	% RE	% Recovery
MET	50	50.37 ± 1.49	0.74	100.74
TEL	40	39.56 ± 0.73	−1.310	98.90
AML	5	4.96 ± 0.07	−0.80	99.20
Standard addition method				
MET	25	24.53 ± 0.37	−1.88	98.12
	50	49.57 ± 0.63	−0.86	99.14
	75	73.58 ± 1.07	−1.89	98.11
	Across Mean			98.46
	% RSD			0.89
TEL	20	19.66 ± 0.35	−1.70	98.30
	40	39.61 ± 0.51	−0.98	99.03
	60	58.92 ± 0.79	−1.80	98.20
	Across Mean			98.51
	% RSD			1.09
AML	2.5	2.46 ± 0.05	−1.6	98.40
	5	5.06 ± 0.07	1.20	101.20
	7.5	7.40 ± 0.14	−0.21	98.70
	Across Mean			99.43
	% RSD			0.72

MET: metoprolol; TEL: telmisartan; AML: amlodipine; SD: standard deviation; %RSD: percentage relative standard deviation; %RE: percentage relative error.

## Data Availability

The data generated during this work are included in the manuscript and submitted as Supplementary Materials.

## Supplementary Materials

The following supporting information can be downloaded at https://www.mdpi.com/article/10.3390/molecules29051087/s1. Table S1: Box–Behnken design for MET, TEL, and AML HPLC method with resolution values; Figure S1: Calibration curves for MET, TEL, and AML; Figure S2: Chromatograms of standard and sample solutions; Figure S3: Greenness AGREE report for the proposed HPLC method; Figure S4: Whiteness report for the proposed HPLC method.
