# Supplementary material for: Design of Experimental Approach for Development of Rapid High Performance Liquid Chromatographic Process for Simultaneous Estimation of Metoprolol, Telmisartan, and Amlodipine from Formulation: Greenness and Whiteness Evaluation
Source: Molecules. 2024 Feb 29;29(5):1087. doi: 10.3390/molecules29051087 (PMC10934861; doi:10.3390/molecules29051087)
Supplement: Supplementary file 1 [file molecules-29-01087-s001.zip › molecules-2834018-supplementary (1).pdf]

Design of experiment approach for development of rapid High Perforance Liquid Chromatographic process for simultaneous estimation of metoprolol, telmisartan and amlodipine from formulation: Greenness and whiteness evaluation

Mahesh Attimarad [1,*] , Mohammed Jassim Alali [1], Hussain Ali Alali [1], Dana Hisham Alabdulmuhsin [1], Aljohara Khalid Alnajdi [1], Katharigatta Narayanaswamy Venugopala [1, 2], and Anroop B. Nair [1]

Table S1. Box Behnken design for MET, TEL and AML HPLC method with resolutions

Standard	Run	X1 A:pH of buffer	X2 B:Acetonitile percent	X3 C:Flow rate	Resolution between 1st and 2nd peaks	Resolution between 2nd and 3rd peaks
12	1	4.5	45	1.2	04.66	14.97
5	2	3	40	0.8	06.47	07.44
14	3	4.5	40	1	10.27	19.53
1	4	3	35	1	09.74	08.46
10	5	4.5	45	0.8	05.74	04.31
6	6	6	40	0.8	08.27	07.60
16	7	4.5	40	1	10.23	19.12
17	8	4.5	40	1	10.00	19.40
4	9	6	45	1	06.91	07.53
3	10	3	45	1	14.43	05.74
2	11	6	35	1	14.43	05.74
9	12	4.5	35	0.8	02.77	21.60
11	13	4.5	35	1.2	10.17	19.63
15	14	4.5	40	1	10.05	19.44
13	15	4.5	40	1	09.96	19.39
8	16	6	40	1.2	07.38	06.75
7	17	3	40	1.2	03.79	05.70

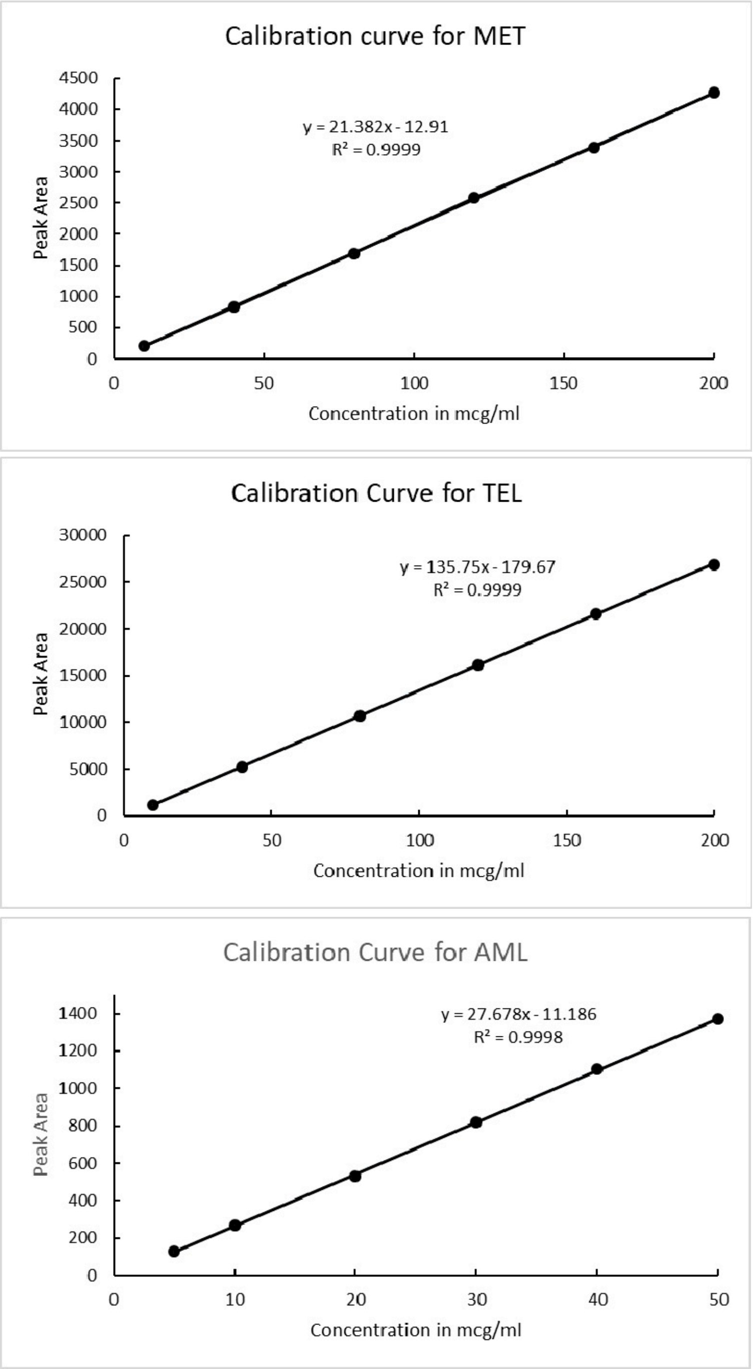

Figure S1: Calibration curves for MET, TEL and AML

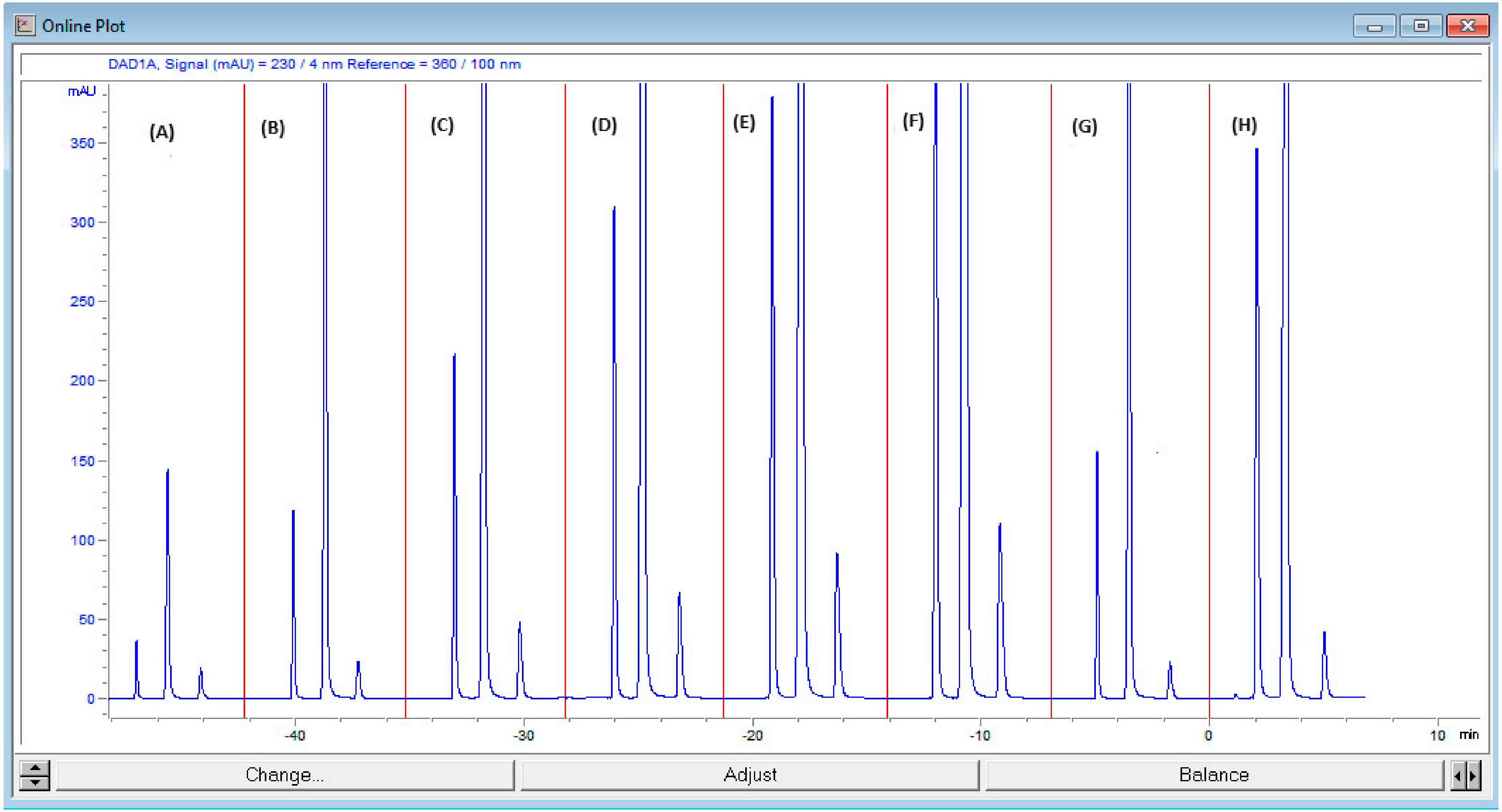

Figure S2: HPLC chromatogram of standard (A-F) and formulation (G, H) solutions of MET (10 – 200 µg/ml), TEL (10 – 200 µg/ml), and AML (5 – 50 µg/ml). MET+TEL+AML in µg/ml : 10+10+5 (A); 40+40+10 (B); 80+80+20 (C);120+120+30 (D); 160+160+40 (E); 200+200+50 (F); Formulation 50+40+5 (G); 150+120+15 (H)

Analytical Greenness report sheet

24/12/2023 18:20:59

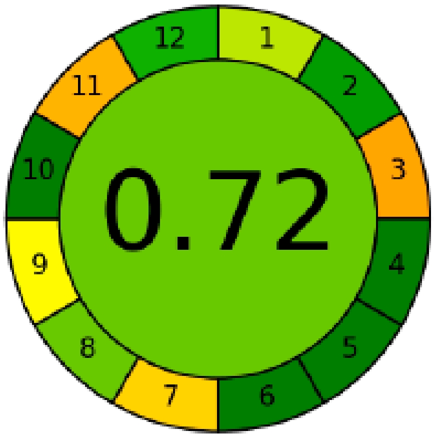

Criteria	Score	Weight
1. Direct analytical techniques should be applied to avoid sample treatment.	0.6	2
2. Minimal sample size and minimal number of samples are goals.	0.88	2
3. If possible, measurements should be performed in situ.	0.33	2
4. Integration of analytical processes and operations saves energy and reduces the use of reagents.	1.0	2
5. Automated and miniaturized methods should be selected.	1.0	2
6. Derivatization should be avoided.	1.0	2
7. Generation of a large volume of analytical waste should be avoided, and proper management of analytical waste should be provided.	0.42	2
8. Multi-analyte or multi-parameter methods are preferred versus methods using one analyte at a time.	0.72	2
9. The use of energy should be minimized.	0.5	2
10. Reagents obtained from renewable sources should be preferred.	1.0	2
11. Toxic reagents should be eliminated or replaced.	0.36	2
12. Operator's safety should be increased.	0.8	2

Figure S3: Greenness AGREE report for the proposed HPLC method

Step 1: Enter the names of methods to the red table (only)

Step 2: Enter the scores to all gray columns in the red, green and blue table, be objective and critical as much as possible. To simplify this, enter the known parameters to the white columns.

Step 3: Analyze the results of assessment presented in the tables below, and in the chart to the right.

RED PRINCIPLES (analytical performance)			R1: Scope of application	R2: LOD and LOQ			R3: Precision			R4: Accuracy		
	Method number	Method name	0-100	LOD	LOQ	0-100	RSD% (repeatability)	RSD% (reproducibility)	0-100	Relative error (%)	Recovery (%)	0-100
	1	HPLC Method	100	0.11-2.60	0.33-7.88	90	0.99-1.91	1.19-1.77	100	0.50-1.82	98.18%-101.30%	100

GREEN PRINCIPLES (green chemistry)			G1: Toxicity of reagents (impact and biodegradation)		G2: Amount of reagents and waste			G3: Consumption of energy and other media	G4: Direct impacts (safety, use of animals and GMOs)			
	Method number	Method name	Total number of pictograms	0-100	Reagent consumption	Waste production	0-100	1-100	Occupational hazards	Safety of users (0-100)	Use of animals (0 if no, 1 if yes)	Use of GMO (0 if no, 1 if yes)
	1	HPLC Method	Toxic solvents	75	More than 10 ml	More than 10 ml	75	85	Hazardus	75	0	0

BLUE PRINCIPLES (practical side)			B1: Cost-efficiency		B2: Time-efficiency		B3: Requirements			B4: Operational simplicity		
	Method number	Method name	Total cost	0-100	Speed of analysis	0-100	Sample consumption	Sample consumption (0-100)	Other needs: advanced instruments, skills, facilities (0-100)	Miniaturization (0-100)	Integration and automation (0-100)	Portability (0-100)
	1	HPLC Method	20 USD /sample	80	8 sample /hour	90	in micrograms	90	90	90	90	90

Figure S4: Whiteness calculation for the proposed HPLC method
